# A58 LOW TTG-IGA ASSOCIATED WITH ISOLATED BULB PATHOLOGY IN PEDIATRIC CELIAC DISEASE: IMPLICATIONS IN A NO-BIOPSY APPROACH ERA

**DOI:** 10.1093/jcag/gwae059.058

**Published:** 2025-02-10

**Authors:** Q Wang, P Jantchou, M Dirks, S Lavoie, L Oligny, D Dal Soglio, N Patey

**Affiliations:** Centre Hospitalier de l’Université de Montréal, Montreal, QC, Canada; Centre Hospitalier Universitaire Sainte-Justine, Montreal, QC, Canada; Centre Hospitalier Universitaire Sainte-Justine, Montreal, QC, Canada; Centre Hospitalier Universitaire Sainte-Justine, Montreal, QC, Canada; Centre Hospitalier Universitaire Sainte-Justine, Montreal, QC, Canada; Centre Hospitalier Universitaire Sainte-Justine, Montreal, QC, Canada; Centre Hospitalier Universitaire Sainte-Justine, Montreal, QC, Canada

## Abstract

NOT PUBLISHED AT AUTHOR’S REQUEST

**Funding Agencies**: None

